# Mortality salience and helping intentions: mediating role of search for meaning and moderating role of negotiable fate

**DOI:** 10.3389/fpsyt.2025.1507212

**Published:** 2025-03-28

**Authors:** Baorui Chang, Jiaxin Cheng, Jiandong Fang, Zhaohua Lyu

**Affiliations:** ^1^ Department of Psychology, School of Education Science, Guangxi Normal University, Guilin, China; ^2^ College Student Mental Health Education Center, Guangxi Normal University, Guilin, China

**Keywords:** mortality salience, helping intentions, search for meaning, negotiable fate, terror management theory

## Abstract

**Introduction:**

This study explores the “altruism born of suffering” hypothesis, which suggests that adversity motivates individuals to help others, potentially mediated by their search for meaning. Grounded in terror management theory, it investigates how mortality salience influences helping intentions and examines the moderating role of negotiable fate, a key destiny view in Chinese culture. Compared to conquering fate and fatalism, negotiable fate is hypothesized to enhance helping intentions when mortality is salient.

**Methods:**

Two experimental studies were conducted. Study 1 tested the mediating role of search for meaning in the relationship between mortality salience and helping intentions. Study 2 examined whether negotiable fate beliefs moderate this relationship, specifically strengthening the effect of mortality salience on helping intentions.

**Results:**

Study 1 found that mortality salience positively predicts helping intentions, fully mediated by search for meaning. Study 2 showed that negotiable fate moderates this relationship, with mortality salience significantly enhancing helping intentions when negotiable fate is salient. These findings highlight the role of negotiable fate in shaping prosocial behavior under mortality reminders.

**Discussion:**

The results emphasize the importance of fostering search for meaning and negotiable fate beliefs to promote helping intentions when mortality is salient. This research advances understanding of how cultural beliefs and existential concerns interact to drive altruistic behavior, suggesting practical interventions to encourage prosocial actions in mortality-salient contexts.

## Introduction

According to Terror Management Theory (TMT), humans can be aware of their own vulnerability and inevitability of death, which produces overwhelming anxiety and fear (1) .In order to alleviate this anxiety and fear, individuals will adopt the maintenance of their own original culture, defensive means such as worldview, boosting self-esteem, or promoting intimacy. Covid-19, as an ongoing global health crisis, undoubtedly stands as one of the most severe disasters humanity has faced in the past few decades (2). Whether it be through quarantine measures, the proliferation of pandemic-related information, or the lingering effects of COVID-19, individuals are constantly confronted with mortality salience, exerting a persistent influence on their mental health (3). Research focusing on mortality salience during the pandemic has garnered considerable attention, contributing empirical insights into the positively oriented terror management theory (TMT). This theory posits that individuals’ responses to mortality salience are not inherently negative, as studies have found that mortality salience induction can increase charitable intentions (4) and that crises often elicit prosocial behaviors (5). For instance, earthquake victims exhibit increased engagement in volunteering activities such as blood donation and rescue efforts (6); laboratory studies have shown that mortality salience primes can enhance charitable behaviors (7); and field studies reveal that participants passing by funeral homes exhibit more positive attitudes towards charitable organizations (8). However, limited research has delved into the underlying mechanisms linking mortality salience to prosocial intentions. This paper seeks to preliminarily explore such mechanisms based on the terror management theory.

Furthermore, research on mortality salience (MS) and prosocial behavior has yielded mixed findings. While some studies report increased prosocial behavior under MS (8, 9), others find no effect or even decreased prosocial behavior, for instance, research has shown that MS decreased compassion toward a person with physical disability among male participants (10). These inconsistencies may stem from cultural factors and individual beliefs, such as negotiable fate (11, 12). The COVID-19 pandemic represents a unique and salient mortality trigger, differing from traditional MS manipulations in its scale, immediacy, and real-world impact. This study aims to address these unresolved issues by examining how COVID-19 as a global mortality threat influences prosocial behavior.

Negotiable fate is more prevalent in Asian cultures, where individuals believe they can negotiate with fate to achieve a better life. Cross-cultural studies have shown that negotiable fate is significantly more common in Asian countries compared to Western cultures (11). For example, in China and India, negotiable fate is associated with more positive thinking styles, constructive coping strategies, and higher self-esteem (11). In contrast, Western cultures tend to emphasize individual control over fate, which may lead to different responses to mortality salience. These cultural differences highlight the importance of considering negotiable fate as a moderating factor in the relationship between mortality salience and prosocial behavior. Cheng et al. (13) suggest that negotiable fate is linked to positive psychological outcomes, such as higher self-esteem, which can reduce death anxiety. As a result, individuals who believe in negotiable fate may experience less anxiety and have a reduced need to enhance self-esteem through pro-social activities.

In contrast, those who are less likely to believe in negotiable fate are less protected by the psychological advantage of negotiable fate (14), and therefore, they are more vulnerable to perceived threats in death-related constraints, so they have a stronger motivation to seek self-esteem enhancement through pro-social behavior.

Under the condition of mortality salience, individuals who hold negotiable fate belief are more likely to reflect on the meaning in life brought about by mortality salience. The novelty of this study resides in adopting a micro-level approach centered on the concept of fate as the entry point, exploring the moderating effect of mortality salience on the intention to help others within the unique sociocultural framework of China.

### Mortality salience and the helping intentions

Mortality Salience, the central notion in Terror Management Theory (TMT), pertains to the awareness of one’s inevitable mortality. According to TMT, humans with advanced cognitive capabilities are aware of their own fragility and the inevitability of mortality. To manage this fear, individuals employ cultural worldviews as a defensive mechanism (15, 16). They give more positive evaluations to people who support their cultural worldview and more to people who don’t negative evaluation in order to consolidate and hold on to their beliefs (1). This means that individuals seek to derive sense of value and self-esteem within the cultural context they belong to. This enables them to feel significant and purposeful in the world. This approach helps them cope with the anxiety stemming from the undeniable reality of their mortality (17). Participation in prosocial activities is an important source that individuals rely upon to strengthen their self-esteem as prosocial causes promote terror management (18), for instance, researchers reported that mortality salience tends to encourage people to offer donations to domestic charity and help to others (18), following mortality salience prime, people behaved more generously when splitting money between themselves and an anonymous partner (19), because engaging in actions that benefit the society meets the shared standards and social norms of most cultures and mortality salience raises the social value of prosocial behaviors (20), thereby enhancing personal value and alleviating death-related anxiety, leading to death transcendence (21).

In most sociocultural settings, prosocial behaviors conform to cultural norms and values (22). When confronted with mortality salience, individuals often engage in helping behaviors to alleviate their fear of death. Empirical studies support this viewpoint (8, 23). For instance, laboratory studies have shown that after being induced with mortality salience, people exhibit more charitable behavior (24). After being presented with images related to death and asked to answer two classic open-ended questions related to death, the experimental group was tested to show more charitable behavior (25).

### The mediating role of search for meaning

What, then, is the underlying mechanism through which mortality salience triggers prosocial intentions? The Shattered Assumption Theory posits that mortality shatters people’s fundamental assumptions about a benevolent world, necessitating the reconstruction of meaning in life (26). The Meaning Making Theory (MMT) further suggests that when individuals encounter traumatic events that disrupt their overarching belief systems, they reassess those events and construct new meanings (27). In other words, when confronted with life-threatening situations, individuals develop psychological need to seek meaning in life (28). Further, some philosophers have argued that thinking of death is useful, which motivates us to live better. For example, Heidegger said that contemplation of death helps us achieve meaning in life and freedom from the fear of death (29). Laboratory studies have revealed that under conditions of mortality salience, individuals engage in cultural worldview defenses as a means of search for meaning (15, 16). This intense motivation to seek meaning in life prompts individuals to engage in behaviors that strengthen social connections (30). Heine et al. propose that when individuals contemplate or search for meaning, they are more inclined to engage in prosocial behaviors (31). Experimental research has found that individuals with a tendency to seek meaning in life exhibit higher levels of prosocial intentions (32), report stronger desires for social engagement, such as engaging in eco-friendly behaviors (33), and donate more money to charitable organizations (32). Consequently, during the process of search for meaning, individuals often demonstrate stronger prosocial intentions (34).

According to Terror Management Theory (TMT), mortality salience triggers existential anxiety, leading individuals to seek ways to alleviate this anxiety. One such way is through the search for meaning, which involves reconstructing a sense of purpose and significance in life (30). The Meaning Maintenance Model (MMT) further suggests that when individuals encounter threats to their meaning systems, such as mortality salience, they engage in behaviors that restore meaning, including prosocial behavior (30). While other constructs, such as self-esteem enhancement, may also play a role in mitigating existential anxiety, the search for meaning is uniquely suited to explain prosocial responses to mortality salience. Prosocial behavior not only reinforces cultural worldviews but also provides individuals with a sense of purpose and significance, thereby addressing the existential threat posed by mortality salience (35). Therefore, we propose that search for meaning mediates the relationship between mortality salience and helping intentions.

Baumeister’s Deficit Correcting Hypothesis holds that the motivation for seeking meaning in life comes from the lack of meaning in life experience (36). Therefore, when facing death threats, Individuals have psychological needs to find meaning in life (28), and prosocial behaviors just meet this need. Relevant studies have found that mortality salience prompts people to have a strong motivation to seek meaning in life, and people make more prosocial behaviors to experience more sense of meaning in life (37). In summary, individuals exposed to mortality salience experience an increased need to restore meaning in life, which, in turn, elevates their willingness to contribute to society (32).

While search for meaning is a key mediator in the relationship between mortality salience and helping intentions, other mechanisms, such as self-esteem, social connectedness, and existential anxiety, may also play a role. For example, prosocial behavior may enhance self-esteem, which buffers against death anxiety (1). Similarly, mortality salience may increase the desire for social bonds, leading to prosocial behavior as a way to strengthen social connections (30). However, search for meaning is particularly relevant in the context of mortality salience, as it addresses the existential need to find purpose and significance in life, which is directly threatened by the awareness of death. Therefore, while other mediators may contribute to prosocial responses, search for meaning provides a unique and comprehensive explanation for the observed effects. Therefore, we propose Hypothesis 1: Search for meaning mediates the relationship between mortality salience and helping intentions.

### The moderating role of negotiable fate

Many studies have shown that the concept of fate is significantly influenced by culture, and the individualism of individual struggle is advocated in the West background, individuals are more inclined to believe that their own efforts can overcome the arrangement of fate, which is called conquerable fate. In the context of Eastern culture, individuals are more inclined to believe in the control of fate over life, which is called fatalistic fate (38). However, in the tussle between individual initiative and fate, some researchers also proposed a third kind of fate, negotiable fate. Individuals who hold this kind of fate not only accept that individuals cannot completely and directly control fate, but also accept that individuals cannot control fate directly. At the same time, they believe that they can negotiate with their fate to attain a better life to a certain extent (39).

More specifically, negotiable Fate refers to the individual’s attribution of uncontrollable and predetermined facts to their own destiny, acknowledging the constraints imposed by fate while simultaneously believing that one can negotiate with fate to a certain extent and strive to create meaning in life (39, 40). As a positive psychological belief, negotiable fate exerts positive effects on cognition, emotional experiences, and coping abilities (41). Individuals with a high level of negotiable fate advocate pursuing personal goals and, despite the limitations imposed by fate, adopt proactive coping strategies (42). For instance, those with a strong negotiable fate belief demonstrate greater perseverance, optimism, and fewer negative emotions when faced with adversity (43). Furthermore, individuals who possess high levels of hope, optimism, and positive emotions tend to have stronger prosocial intentions (44, 45). Direct evidence for this has been provided by research, such as a study by Liem and colleagues, which found that individuals with a high negotiable fate belief were more likely to donate money to street children to improve their lives. While acknowledging that the plight of street children may be predetermined, they believe that donations can help improve these children’s living conditions (46). Based on this, we propose Hypothesis 2: Negotiable fate belief moderates the relationship between mortality salience and helping intentions.

The Terror Management Theory (TMT) posits that when individuals confront mortality salience, they seek meaning and create value within their respective socio-cultural contexts to manage the fear of mortality (17). Helping others not only fulfills people’s need for pursuing meaning in life but also generates societal value (47). Therefore, the motivation to find meaning in life enhances individuals’ willingness to help others. Mortality being an inevitable part of human existence, those who hold a negotiable fate belief, confronted with the unalterable fact of mortality, still choose to believe that they can negotiate with fate, exert personal agency, and achieve life goals through individual effort (39). Based on a review of the literature, this study hypothesizes that individuals with negotiable fate belief will exhibit a higher willingness to help under conditions of mortality salience.

**Figure 1:**
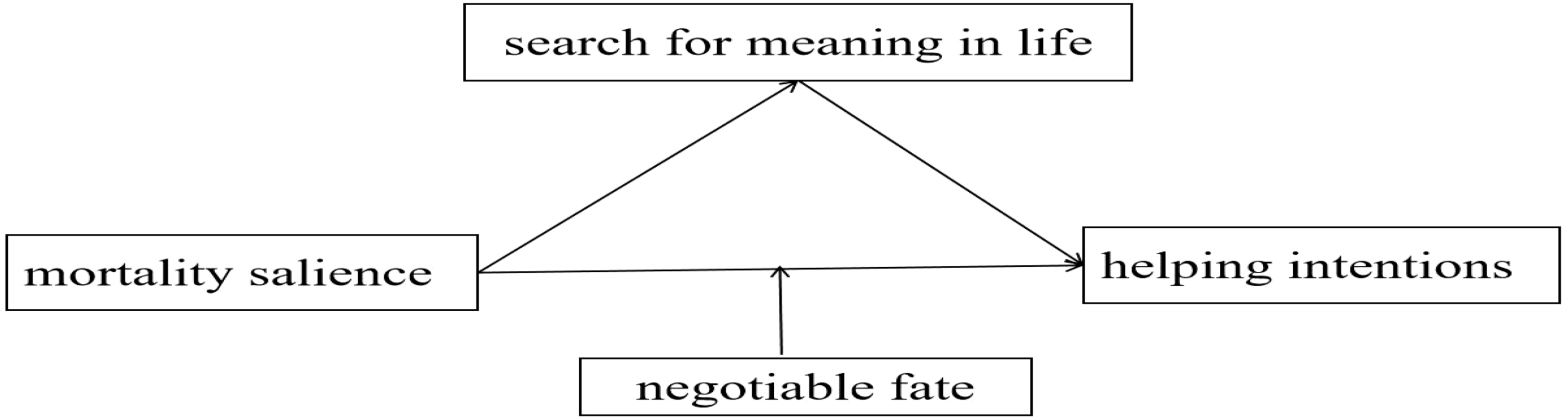
Theory model.

This study will employ two experimental investigations to examine the mediating role of search for meaning between mortality salience and helping intentions, as well as the moderating role of negotiable fate in this relationship. The theoretical model is illustrated in **Figure 1**.

## Measure

### Study 1: the impact of mortality salience on helping intentions: the mediating role of search for meaning

#### Participants

Using G*power 3.1, we estimated the required sample size to achieve a statistical power of 1-β = 0.80, with an alpha level of 0.05 and a medium effect size of 0.25. A total of 128 participants were needed. However, 250 participants were recruited for this study. After excluding 4 cases due to failed priming, 246 valid data sets were obtained (*M*age = 18.75, *SD* = 0.94; 39 males, 207 females), effective rate was 98.40%.

#### Study procedure

A single-factor between-subjects experimental design was employed, with participants randomly assigned to either the mortality salience group or the dental pain salience group so as to control the sample differences under different experimental conditions and avoid the confusing effects of individual differences on the results. Taking the toothache group as the control group is the practice of referring to previous studies. Toothache and death are too painful to let people doubt about their life.

Participants were instructed to perform a corresponding imagery task (imagining mortality *vs*. imagining dental pain). Following this, they completed a delayed distraction task before assessing their levels of search for meaning and helping intentions. Finally, participants completed demographic questions.

#### Experimental material

##### Manipulation and verification of the independent variable

###### Manipulating mortality salience

We used two open-ended questions from the classic research paradigm of mortality salience (18) to guide participants in responding in writing. Following previous studies, participants were then required to complete three 11-point Likert-scale manipulation check items (mortality thought accessibility, fear, and unpleasantness) (48) to verify the manipulation.

##### Delayed distraction task

Given that the enhancement of mortality salience effects occurs approximately 15 minutes or after two to three intervening tasks following the mortality salience manipulation (36), we followed previous research by adopting the Positive and Negative Affect Schedule (PANAS) developed by Watson et al. (1988) (49) and revised by Huang and Yang (2003) (50) in China, along with a number puzzle game, as materials for the delayed distraction task following the mortality salience manipulation.

##### Meaning in life scale

The Meaning in Life Scale adopted the dimension of search for meaning from the revised Chinese version of the Meaning in Life Questionnaire by Wang (2016) (12), consisting of 5 items. A 7-point Likert scale was used (1 = “Completely disagree”, 7 = “Completely agree”), with the total mean score serving as the statistical indicator. Higher scores indicated a stronger tendency to search for meaning. The Cronbach’s α coefficient for the Meaning in Life Scale in this study was 0.84.

##### Measurement of helping intentions in hypothetical scenarios

Drawing from previous research paradigms (51), two hypothetical scenario questions were developed to measure helping intentions. Scenario 1 assessed the individual’s willingness to donate money, while Scenario 2 assessed the willingness to donate material goods. Donation of money and donation of materials are two kinds of prosocial behaviors, which need to sacrifice individual’s own interests. We hypothesized that mortality salience can promote the influence of these two kinds of prosocial behaviors under the epidemic situation. A 7-point Likert scale was employed, with the total mean score serving as the statistical indicator. Higher scores indicated higher helping intentions.

#### Results

##### Manipulation check for mortality salience

Independent samples t-tests revealed significant differences in mortality thought accessibility between the mortality salience group (*M*=5.08, *SD*=1.88) and the dental pain control group (*M*=1.82, *SD*=1.55), *t* (244) = -14.87, *p* < 0.001, |Cohen’s d| = 1.89. However, there were no significant differences in fear between the mortality salience group (*M*=4.06, *SD*=2.41) and the dental pain group (*M*=4.15, *SD*=2.84), *t* (244) = 0.29, *p* = 0.772, |Cohen’s d| = 0.03, or in unpleasantness between the mortality salience group (*M*=3.75, *SD*=2.36) and the dental pain control group (*M*=3.20, *SD*=2.48), *t* (244) = -1.76, *p* = 0.079, |Cohen’s d| = 0.23. These results indicate that the manipulation was effective.

##### Correlation analysis

The means, standard deviations, and correlation matrix for the main core variables are presented in **Table 1**. Since gender was significantly positively correlated with the dependent variable, it was included as a control variable in subsequent analyses.

**Table 1:** Correlation matrix of main variables.

Main variables	*M* ± *SD*	1	2	3	4
1.Group	—	—			
2.Gender	—	0.08	—		
3.Family Origin	—	0.07	-0.09	—	
4.Search for Meaning	5.07 ± 1.03	0.15^*^	0.15^*^	0.03	—
5.Helping Intentions	5.65 *±* 1.02	0.09	0.26^**^	-0.02	0.38^**^

**p<0.01, *p<0.05; Categorical variable virtualization: Group:1=mortality salience group, 0=dental pain group; Gender:1=male, 0=female; Family origin:1=city, 0=village.

#### Analysis of the mediating effect of search for meaning

The groups were dummy-coded, with 1 representing the mortality salience group and 0 representing the dental pain salience group. The PROCESS Model 4 macro program for SPSS, was employed to examine the mediating role of search for meaning between group membership and helping intentions. In this study, gender was included as a control variable. As shown in **Table 2**, the bias-corrected percentile Bootstrap test revealed that the indirect effect of search meaning was 0.09, with a 95% confidence interval of [0.01, 0.19]. The mediating effect accounted for 64.3% of the total effect (0.14), indicating that search for meaning fully mediated the relationship between mortality salience and helping intentions.

**Table 2:** Simple mediation effect analysis.

Main variables	Equation 1 (Dependent variable: Search for meaning)				Equation 2 (Dependent variable: Helping intentions)			
	β	*SE*	*t*	Boot 95%*CI*	β	*SE*	*t*	Boot 95%*CI*
Group	0.28	0.13	2.12^**^	[0.02,0.54]	0.05	0.12	1.16	[-0.17,0.28]
SFM					0.34	0.06	5.93^***^	[0.22,0.45]
*R^2^*	0.04				0.43			
*F*	4.97^**^				18.97^***^			

^***^ *p*<0.001,^**^ *p*<0.01, ^*^ *p*<0.05; Group, mortality salience/dental pain; SFM, search for meaning; HI, helping intentions.

#### Summary

Study 1 manipulated mortality salience and found that search for meaning fully mediated the impact of mortality salience on helping intentions, thus validating Hypothesis 1. However, the question remains regarding the boundary conditions under which mortality salience influences helping intentions. In Study 2, we will simultaneously manipulate mortality salience and negotiable fate, further exploring the moderating role of negotiable fate in the relationship between mortality salience and helping intentions, and re-examining the mediating role of search for meaning.

### Study 2: the mediating role of search for meaning and the moderating role of negotiable fate

#### Participants

This study employed a two-factor between-subjects experimental design. Using G*power 3.1, we estimated that a sample size of 211 participants would achieve a statistical power of 1-β=0.80 with α=0.05 and a medium effect size of 0.25. A total of 230 participants were recruited, with 7 excluded due to failed manipulations or extreme values exceeding ±3 standard deviations. The final valid sample consisted of 223 participants (*M*age = 19.44, *SD* = 1.67; 59 males, 164 females), yielding an effective rate of 96.95%. Participants were randomly assigned to six groups, with each group containing no fewer than 35 participants.

#### Research design and procedures

A two-factor between-subjects experimental design was adopted: 2 (priming type: mortality salience *vs*. dental pain salience) × 3 (type of beliefs about fate: neutral control group *vs*. negotiable fate *vs*. conquerable fate). Participants were randomly assigned to one of the six groups. The priming procedures for the independent variables were identical to those in Study 1, with participants performing an imagination-writing task based on experimental instructions.

Considering that the effect of mortality salience typically peaks approximately 15 minutes after its induction, according to Au (2008), the manipulation of negotiable fate was implemented as a delayed distraction task subsequent to the mortality salience manipulation. it is presumed to focus on the core concept of negotiable fate, potentially incorporating statements or scenario descriptions illustrating how individuals negotiate with fate when confronting life challenges while retaining autonomy within an established fate framework. For instance, participants might encounter narratives such as, “Despite being born into poverty, an individual, through personal effort, altered their life path to some extent, achieving a compromise and progression with fate,” to facilitate their consideration and acceptance of the concept of negotiable fate.

For the neutral control group, general and neutral information or tasks unrelated to fate were provided to avoid influencing participants’ fate beliefs, serving as a baseline for comparing the effects of the negotiable fate and conquerable fate groups. The stimulus materials for the conquerable fate group likely emphasized that individuals can fully transcend fate’s limitations through strong will and effort, presenting examples or perspectives of personal struggle triumphing over fate’s arrangements, such as, “Where there is a will, there is a way” to prompt participants to consider and identify with the belief in conquerable fate.

Following this, participants were randomly exposed to different types of beliefs about fate (neutral control group, negotiable fate, or conquerable fate), various potential variables among participants in different groups were ensured to be as balanced as possible through randomization, thus reduce result bias due to unbalanced grouping. Finally, they completed the measures of search for meaning, helping intention, and demographic variables.

#### Measure

The mortality salience priming was identical to that in Study 1.

To prime negotiable fate, we considered that the enhancement of mortality salience effects typically occurs approximately 15 minutes after the mortality salience prime. Therefore, the manipulation of the moderating variable was administered as a delayed distraction task following the mortality salience manipulation. We translated the negotiable fate prime materials developed by Au (2008) (52) and finalized the Chinese version through team discussions.

Manipulation Check.

The negotiable fate checklist, also developed by Au (2008) (52), was used. Responses were scored on a 7-point Likert scale, with 1 representing “strongly disagree” and 7 representing “strongly agree.” The measures of search for meaning and helping intentions were identical to those in Study 1.

#### Results

##### Manipulation checks

###### Mortality salience manipulation check

An independent samples t-test revealed a significant difference in mortality-related thoughts between the mortality salience group (*M*=5.62, *SD*=2.15) and the dental pain control group (*M*=2.40, *SD*=2.19), *t* (221) = -11.07, *p* < 0.001, |Cohen’s d| = 1.48. However, there was no significant difference in fear between the mortality salience group (*M*=4.98, *SD*=2.58) and the dental pain salience group (*M*=5.36, *SD*=3.15), *t* (221) = 0.99, *p* = 0.322, |Cohen’s d| = -0.13. Similarly, there was no significant difference in unpleasant feelings between the mortality salience group (*M*=4.47, *SD*=2.35) and the dental pain control group (*M*=3.83, *SD*=2.74), *t* (221) = -1.87, *p* = 0.063, |Cohen’s d| = 0.25. These results indicate that the manipulation was effective.

##### Negotiable fate manipulation check

A one-way ANOVA revealed a marginally significant difference in negotiable scores between the negotiable fate group (*M*=6.11, *SD*=0.93), the conquerable fate group (*M*=5.80, *SD*=0.92), and the neutral control group (*M*=5.81, *SD*=0.85), *F* (2,220) = 3.00, *p* = 0.052, η_p_² = 0.03. These results suggest that the manipulation was effective, albeit marginally, in priming negotiable fate beliefs.

##### Examination of mediating and moderating effects

We used PROCESS Model 5 to test the combined effects of mediation and moderation. To ensure robustness and exclude alternative mechanisms, we conducted additional analyses using PROCESS Model 7 and Model 14. Consistent with prior research, indicating gender differences in prosocial behavior (53), we examined the mediating effect of the search for meaning and the moderating role of negotiable fate in the relationship between mortality salience and helping intentions.

As shown in **Table 3**, the results of the moderated mediation analysis revealed that the group significantly predicted the search for meaning (β=0.24, *SE*=0.12, *p*=0.045). The search for meaning marginally significantly predicted helping intentions (β=0.13, *SE*=0.07, *p*=0.066), whereas the group did not significantly predict helping intentions directly (β=−0.10, *SE*=0.21, *p*=0.608). Therefore, the search for meaning fully mediated the relationship between mortality salience and helping intentions. When fate beliefs were virtualized, using the neutral control group as the baseline, we found that the interaction between negotiable fate beliefs and group significantly predicted helping intentions (β=0.57, *SE*=0.29, *p*=0.047), indicating that negotiable fate beliefs moderated the relationship between the group (mortality/dental pain) and helping intentions.

**Table 3:** Tests of moderated mediating effects.

Variables	Equation 1 (Dependent variable: Search for meaning)				Equation 2 (Dependent variable: Helping intentions)			
	β	*SE*	*t*	Boot 95%*CI*	β	*SE*	*t*	Boot 95%*CI*
Group	0.24	0.12	2.03	[0.01, 0.47]	-0.10	0.21	-0.49	[-0.52, 0.32]
SFM					0.13	0.07	1.88	[-0.01, 0.27]
NF					-0.08	0.22	-0.39	[-0.52, 0.35]
CF					-0.08	0.23	-0.34	[-0.53, 0.37]
Group*NF					0.57	0.29	1.99	[0.01, 1.15]
Group*CF					0.21	0.31	0.67	[-0.40, 0.82]
*R^2^*	0.03				0.14			
*F*	3.36^*^				4.88^***^			

Note: ^***^ *p*<0.001,^**^ *p*<0.01, ^*^ *p*<0.05; Group, mortality salience/dental pain; SFM, search for meaning; NF, negotiable fate; CF, conquering fate; HI, helping intentions.

**Figure 2:**
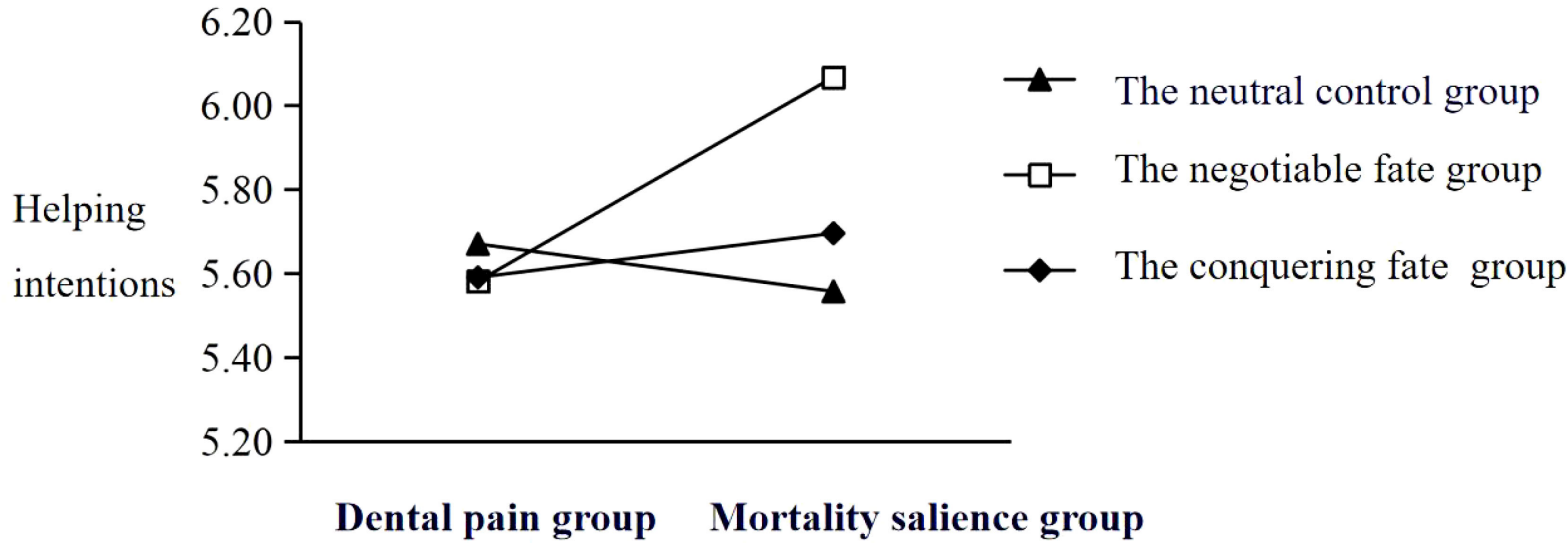
Simple slope.

As depicted in **Figure 2**, further simple slope analysis revealed that under the condition of negotiable fate, mortality salience significantly and positively predicted helping intentions (βsimple=0.46, 95%CI=[0.07, 0.86], *p*=0.019). In contrast, the direct effect of mortality salience on helping intentions was not significant in the neutral control group (β=−0.1, *SE*=0.21, *p*=0.608) or the conquering fate group (β=0.11, *SE*=0.23, *p*=0.659).

#### Summary

In summary, mortality salience exerts its influence on helping intentions through the complete mediation of the search for meaning in life. Both experiments revealed that mortality salience does not directly predict helping intentions, but its direct path is moderated by fate beliefs. For individuals with activated negotiable fate beliefs, mortality salience significantly and positively predicts helping intentions, whereas for those without such beliefs, mortality salience does not directly impact helping intentions.

## Discussion

Through two progressive studies, this paper demonstrates the mediating role of the search for meaning in life between mortality salience and helping intentions, and the moderating effect of negotiable fate beliefs on the direct path from mortality salience to the helping intentions. Specifically, under the condition of activated negotiable fate beliefs, mortality salience significantly and positively predicts helping intentions.

### Mortality salience and helping behavior

The results of this study showed that death mortality has no significant predictive effect on helping behavior, which was inconsistent with previous research results (54), such as, when death became apparent, Israeli soldiers were more likely to help Jews (in-group) than Arabs (out-group) (55). As depicted in the dual process model in TMT, these remedial actions and attitudes aimed at regulating our death anxiety, may or may not be logical and relevant to the threats at hand. Instead, they are determined by the salience of death thoughts in the individuals’ conscious awareness (55). Based on the dual process model, if death thoughts are at the forefront of our awareness, we are more inclined to engage in proximal defenses. As death thoughts recede and hover at the periphery of consciousness after a time delay, we will shift towards distal defenses. Distal defenses are responses that may appear illogical and contradictory to the current threats, but are helpful in alleviating anxiety through the enforcement of our worldview, self-esteem, or relational needs (55). In both studies, the distraction task was performed after the death initiation, and then helping intention was measured, but the interval was short and still in the proximal defense phase, so the predictive effect was not significant. The results were consistent with those of previous studies, which proved that individuals were more inclined to help others immediately after pandemic salience regardless of self-construal (56).

Our findings indicate that mortality salience does not have a significant direct effect on helping behavior. However, it does have a significant indirect effect through search for meaning, suggesting that the total effect of mortality salience on helping behavior is equivalent to the indirect effect. This highlights the importance of search for meaning as a key mechanism driving prosocial responses to mortality salience.

### The mediating role of the search for meaning

The results of both Study 1 and Study 2 indicate that search for meaning fully mediates the relationship between mortality salience and helping intentions, thus confirming Hypothesis 1. This finding offers a possible explanation for “altruism stemming from suffering,” suggesting that individuals who have experienced hardship become more motivated to help others because of their suffering enhances their quest for meaning in life (57). The meaning maintenance model posits that humans are creatures that seek and create meaning (31), and the pursuit of meaning in life serves as the best way to alleviate the fear of mortality (58). Individuals who seek meaning in life tend to engage in prosocial behaviors that reflect cultural values (59). Steger and Kawabata (60) argue that the search for meaning is a process of self-improvement aimed at realizing life’s values and gaining sense of meaning (61). Our findings reveal that the search for meaning among college students in response to mortality salience promotes helping behaviors, highlighting the importance of effectively search for meaning. Helping others is a mature and feasible approach to search for meaning (61). This result not only underscores the close connection between meaning in life and social relationships, but also demonstrates that helping behaviors are an effective way for college students to seek for meaning in life under the influence of mortality salience. As any given culture functions to serve its members with a symbolic description of meaning and value (1), individuals quell their existential anxiety and regain a sense of equanimity by resorting to the “dual-component cultural anxiety buffer” (8), which consists of cultural worldview that represents a meaningful conception of reality, as well as self-esteem that endorses one’s self-view as a valuable member living up to the general and role-specific standards prescribed by the culture (21, 61).

More important, this study adds to the literature on the relationship between mortality salience and helping intention. The results were consistent with previous studies, such as, donate government subsidies or provide free meals to those in need during the COVID-19 pandemic (56, 62). Others also documented caring and helping actions during COVID-19, including providing emotional support to family and friends, making higher donations to emergency funds, and showing greater interest in volunteering for COVID-19-related causes (63).

### Moderating effect of negotiable fate

The novelty of this study lies in constructing a moderated mediation model, which reveals that negotiable fate significantly moderates the path from mortality salience to helping intentions, thereby validating Hypothesis 2. Specifically, participants who were primed with negotiable fate exhibited higher helping intentions under mortality salience compared to those in the neutral control group and the fatalistic control group. This finding aligns with previous research, indicating that individuals with high negotiable fate are better equipped to confront existential adversity (62). For instance, studies have shown that negotiable fate can buffer the relationship between bullying victimization and psychological distress among adolescents (63). Furthermore, individuals with high negotiable fate are more likely to adopt proactive coping strategies, such as engaging in charitable behaviors, to enhance their sense of control (64). Related research also found that new-generation migrant workers with negotiable fate possess favorable adaptation strategies and self-cognition (65), which, in turn, facilitate higher helping intentions (66). Consequently, individuals with negotiable fate are more receptive to the “fate” of mortality and alleviate mortality anxiety by engaging in helping behaviors (44). According to the broaden-and-build theory, individuals with high negotiable fate can better construct enduring and stable social and psychological resources (67). Compared to those in the neutral control and fatalistic groups, participants primed with negotiable fate are more accepting of mortality salience and believe that they can exert maximum effort within their limited lifespan, thereby demonstrating higher helping intentions. This study’s findings suggest that enhancing individuals’ negotiable fate can increase their helping intentions, enabling them to better cope with mortality salience information.

### Cross-scale integration in prosocial behavior research

While our investigation primarily examines the psychological mechanisms underlying the relationship between mortality salience and helping intentions, it is crucial to recognize that prosocial behavior is shaped by multifaceted factors operating across different scales. At the micro level, research in genetics, molecular biology, and neuroscience has significantly advanced our understanding of the biological foundations of prosocial behavior (68). Notably, empirical studies have identified specific genetic markers and neural circuits associated with empathy, altruism, and cooperative behavior (69), suggesting that individual differences in prosocial tendencies may stem from biological predispositions that interact with psychological and environmental factors.

At the macro level, socio-cultural norms, public health conditions, and environmental factors substantially influence prosocial behavior. Cross-cultural research demonstrates that collectivist and interdependent cultural norms significantly enhance cooperative and altruistic behaviors (70). Contemporary global challenges, particularly the COVID-19 pandemic, have further underscored the critical role of prosocial behavior in maintaining social cohesion and collective well-being (71). Additionally, environmental factors, including natural disasters and resource scarcity, have been shown to substantially impact prosocial behavior through their effects on social dynamics and individual motivations (5).

Despite substantial progress at both micro and macro levels, the field continues to face significant challenges in integrating findings across these scales. This limitation represents a critical barrier to understanding the complex interplay of factors influencing prosocial behavior. Future research should prioritize the development of interdisciplinary approaches that synthesize insights from genetics, neuroscience, psychology, sociology, and environmental science (72). Potential research directions could include examining how genetic predispositions interact with cultural norms across varying environmental contexts, or investigating how public health interventions and socio-cultural practices influence the neural and psychological mechanisms underlying prosocial behavior.

In conclusion, while our study contributes to the understanding of psychological mechanisms linking mortality salience to helping intentions, we emphasize the necessity for a more comprehensive approach that integrates findings across multiple scales. Through fostering cross-scale collaboration and interdisciplinary research, we can achieve a more nuanced understanding of the complex determinants of prosocial behavior and develop more effective strategies for promoting social cohesion and well-being in diverse contexts.

### Limitations and future directions

This study explored the mechanism of mortality salience on helping intention and verified the boundary role of negotiable fate, contributing theoretically and providing practical guidance for life education. However, several limitations exist. First, One possible limitation of this study is that our participants are mainly university students. It is a common concern for economic and psychological experiments whether the results from students generalize to other people (73). Researchers have found that students behave less generously than other social members (74, 75), we suggest that ordinary people primed with mortality salience might display more prosocial behaviors. Future studies can be done on participants of different age groups, e.g., adolescents (76) or older adults (77) to examine how the effect of mortality salience differs for people with different remaining life expectancy. In addition, we did not measure the preferences of the two groups prior to priming. Future investigations could provide a more accurate estimate the effects of priming if they can elicit the time preference both before and after the priming. Third, It is true that our sample exhibited a gender imbalance, with 83% of the participants in study 1 and 73% in study 2 being female. Follow-up studies will balance the gender. Finally, it remains unclear whether the priming effect can last for a long time, which would be an interesting and important issue for subsequent research.

## Conclusion

This study examined the mechanism and boundary conditions of mortality salience on helping intention through two studies, yielding the following conclusions:

1. After controlling for gender, mortality salience significantly and positively predicted helping intention through the full mediation of the search for meaning.
2. Negotiable fate moderated the direct path from mortality salience to helping intention. Specifically, for individuals primed with negotiable fate, the direct positive predictive effect of mortality salience on helping intention was significant. Therefore, enhancing individuals’ belief in negotiable fate can facilitate positive coping with mortality salience.

## Data Availability

The original contributions presented in the study are included in the article/supplementary material. Further inquiries can be directed to the corresponding author.
